# Undiagnosed Behçet's Disease Presenting as Fournier's Gangrene in a Young Male

**DOI:** 10.1155/2021/6626909

**Published:** 2021-09-21

**Authors:** Jasmine C. Winyard, Anton Wong, Hala Rashed, John K. Mellon

**Affiliations:** ^1^Department of Urology, Leicester General Hospital, UK; ^2^Department of Histopathology, University Hospitals Leicester, Gwendoline Road, Leicester LE5 4PW, UK

## Abstract

Behçet's disease is rare, especially in the paediatric population. In this case, a healthy 16-year-old made presented with discrete scrotal ulcers and systemic illness. He was found to have Fournier's gangrene and with subsequent investigation was diagnosed with Behçet's disease as an underlying cause. A PubMed search reveals no similar case reports. His only risk factors for Fournier's gangrene was his raised body mass index. His only risk factor for Behçet's disease was his ethnic origin. An understanding of risk factors and epidemiology can raise suspicion of these rare pathologies.

## 1. Introduction

Behçet's disease is a systemic inflammatory disease which is most prevalent in the Middle East, Mediterranean region, and Eastern Asia [1]. It typically presents in the third to fourth decade of life [2]. In the UK, the incidence is 0.64/100000 [3]. Oral ulceration and genital ulceration are common features of Behçet's disease, but genital ulceration is less common in younger patients [4]. We report a rare case of Fournier's gangrene in a young male, complicating underlying Behçet's disease.

## 2. Case Presentation

A 16-year-old male, of Middle Eastern origin, presented as an emergency admission with painful scrotal skin lesions of 3-day duration. He also reported that he had experienced intermittent mouth ulcers for the past year, for which he had used topical aciclovir without seeking medical attention. He was not sexually active.

On examination, he was febrile with a temperature of 38.1°C. His BMI was 31. Scrotal examination revealed two discrete areas of well-demarcated skin necrosis with surrounding erythema. The lesions appeared to be confined to the scrotal skin and did not involve the testes or epididymis. Blood tests revealed a white cell count of 14.5 × 10^9^/L; CRP of 38 mg/L, and a blood glucose of 4.7 mmol/L. A provisional diagnosis of Behçet's disease with a superimposed infection was made.

His temperature settled, and he remained clinically stable following the initiation of intravenous meropenem. Following 48 hours of treatment, however, his clinical condition deteriorated. He became pyrexial (40°C) and tachycardic (140 bpm). His white cell count worsened (WCC 19 × 10^9^/L). A rheumatology opinion was sought, and they agreed that an underlying diagnosis of Behçet's disease was possible. A serology sample was sent for a HLA-B51 assay. Scrotal ultrasound was arranged which showed no abscess but some oedema around the left epididymis. A decision was made to treat with a surgical debridement under general anaesthetic.

**Figure 1:**
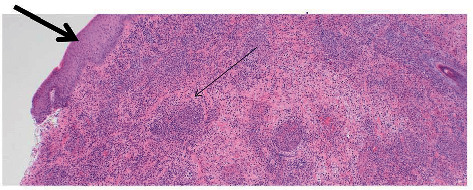
Excised scrotal ulcer and skin from the case described. Stained with haematoxylin-eosin, ×4 magnification. Epidermal ulceration (shown by large arrowheads) with deeper inflammatory infiltrates (shown by small arrowheads). The full histology report described full-thickness necrosis with abscess formation, in keeping with Fournier's gangrene.

Histology of the excised skin lesion showed full thickness necrosis, consistent with Fournier's gangrene (Figure 1). Prior to discharge, the patient was commenced on colchicine on the advice of the rheumatologist, but prior to his raised HLA B51 result. Following discharge from the ward, the urology team reviewed the patient on a number of occasions, and the excision sites have healed well. Further small ulcerations, without necrosis, on the scrotum were noted. The rheumatology team have commenced him on colchicine and prednisolone, and subsequently, methotrexate, for newly diagnosed Behçet's disease. He remains under their care six months later.

## 3. Discussion

Behçet's disease typically presents in the third or fourth decade of life. Patients less than age 16 account for 4-24% of cases [2]. The presence of the HLA-B51 allele in this patient strongly supports the diagnosis, as it is the strongest predisposing genetic trait [5]. The presence of this allele predisposes to an increased risk of genital ulceration, but carries a reduced risk of gastrointestinal involvement.

This patient was of Middle Eastern origin. As Behçet's disease is most prevalent in populations on the old silk trade routes, a suspicion of this disease was raised in this case. The patient had been suffering from oral ulcers for many months which, combined with genital ulceration, is a common presentation of Behçet's disease [6]. Interestingly, the frequency of genital ulcers is lower in the paediatric population compared with adults [4]. The vast majority of patients (87-97%) with Behçet's have oral ulceration at presentation; however, only 55-83% of paediatric patients have genital ulceration [4]. In male Behçet's patients with genital ulceration, the scrotum is the most common location [6]. Typically, genital ulcers are described as looking similar to aphthous ulcers but with a deeper base. Initially, erythematous and raised, the centre then becomes pale with a grey-yellow base [3]. This was not the case in this patient. In this patient, the ulcers were very shallow with a disc-like dark thickening at the surface.

The complication of Fournier's gangrene this patient had is an aggressive and life-threatening condition which requires immediate surgical debridement. It is rare for Behçet's disease to be complicated by Fournier's gangrene. In fact, a PubMed search of “Behçet's AND Fournier's” does not return any reports of this presentation.

This case caused diagnostic uncertainty. In a comorbid patient, who was clinically unwell with scrotal lesions, an immediate diagnosis of Fournier's gangrene would have likely been made. In such cases, a decision to take a patient to theatre would have been straightforward, and a diagnosis of Behçet's disease might have been made at a later stage. The patient had a normal blood glucose level and had no history of scrotal trauma, which both often precede Fournier's gangrene. This patient's only risk factor for Bechet's was his ethnicity, and for Fournier's gangrene, his raised BMI.

This young patient presented in an unusual way, with a synchronous diagnosis of Fournier's gangrene superimposed on Behçet's disease. This case is a reminder that although we must be careful not to stereotype unnecessarily genetic predisposition and therefore racial origin can raise suspicion of the more uncommon pathology. Knowledge of risk factors and epidemiology is therefore useful.

## Data Availability

No data other than personal patient data.
